# Outcome after protected full weightbearing treatment in an orthopedic device in diabetic neuropathic arthropathy (Charcot arthropathy): a comparison of unilaterally and bilaterally affected patients

**DOI:** 10.1186/s12891-016-1357-4

**Published:** 2016-12-29

**Authors:** Niklas Renner, Stephan Hermann Wirth, Georg Osterhoff, Thomas Böni, Martin Berli

**Affiliations:** 1 Orthopädische Klinik Luzern AG, Hirslanden Klinik St.Anna, Luzern, Switzerland; 2 Department of Orthopedics, University of Zürich, Balgrist University Hospital, Zürich, Switzerland; 3 Department of Trauma Surgery, University Hospital of Zürich, Zürich, Switzerland

**Keywords:** Bilateral, Charcot, Diabetic neuropathic arthropathy, Neuroosteoarthroapthy, Infection, Protected weightbearing, Total contact cast, Ulcer

## Abstract

**Background:**

Charcot neuropathic arthropathy (CN) is a chronic, progressive, destructive, non-infectious process that most frequently affects the bone architecture of the foot in patients with sensory neuropathy. We evaluated the outcome of protected weightbearing treatment of CN in unilaterally and bilaterally affected patients and secondarily compared outcomes in protected versus unprotected weightbearing treatment.

**Methods:**

Patient records and radiographs from 2002 to 2012 were retrospectively analyzed. Patients with Type 1 or Type 2 diabetes with peripheral neuropathy were included. Exclusion criteria included immunosuppressive or osteoactive medication and the presence of bone tumors. Ninety patients (101 ft), mean age 60.7 ± 10.6 years at first diagnosis of CN, were identified. Protected weightbearing treatment was achieved by total contact cast or custom-made orthosis. Ulcer, infection, CN recurrence, and amputation rates were recorded. Mean follow-up was 48 (range 1–208) months.

**Results:**

Per the Eichenholtz classification, 9 ft were prodromal, 61 in stage 1 (development), 21 in stage 2 (coalescence) and 10 in stage 3 (reconstruction). Duration of protected weightbearing was 20 ± 21 weeks and 22 ± 29 weeks in patients with unilateral and bilateral CN, respectively. In bilaterally affected patients, new ulcers developed in 9/22 (41%) feet. In unilaterally affected patients, new ulcers developed in 5/66 (8%) protected weightbearing feet and 4/13 (31%) unprotected, full weightbearing feet (*p* = 0.036). The ulceration rate was significantly higher in bilaterally versus unilaterally affected patients with a protected weightbearing regimen (*p* = 0.004). Soft tissue infection occurred in 1/13 (8%) unprotected weightbearing feet and 1/66 (2%) protected weightbearing feet in unilaterally affected patients, and in 1/22 (4%) protected weightbearing feet of bilaterally affected patients. Recurrence and amputation rates were similar across treatment modalities.

**Conclusions:**

Bilateral CN results in significantly more ulcers than unilateral CN and leads to slightly higher soft tissue infections. Protected weightbearing in an orthopedic device can reduce the risk for complications in acute CN of the foot and ankle.

## Background

Charcot neuropathic arthropathy (CN) is a chronic, progressive, destructive, non-infectious process leading to progressive degeneration of a weightbearing joint. It most frequently affects the osseous alignment of the foot and joint alignment in people with sensory neuropathy [1]. The disease was first described in 1868 by Jean-Martin Charcot in the context of tabes dorsalis [2, 3]. Today, diabetes mellitus is the most common cause of peripheral neuropathy and is therefore often associated with the development of CN. From 0.08 to 7.5% of diabetic patients are diagnosed with CN [4]. With the worldwide increase in the prevalence of diabetes, diagnosis and treatment of CN is becoming more important [5].

The main characteristics of CN are atraumatic swelling with redness and warmth, followed by deformation of the foot. Often this occurs in only one foot [6]. The exact pathogenesis of CN is still unclear. However, there are several predisposing factors, such as the retention of vasodilitatory reflexes of the affected foot, upregulation of calcitonin gene related peptide and reduction in bone mineral density [7–9]. Once the disease has been triggered, it progresses to an uncontrolled inflammation. The loss of pain sensation and proprioception combined with repetitive mechanical trauma to the foot leads to fractures and joint dislocations [10–12]. Once a bone has fractured, proinflammatory cytokines such as tumor necrosis factor-α (TNF-α) and interleukin-1β (IL-1β) are released, resulting in increased expression of receptor activator of nuclear factor-kB ligand (RANKL), synthesis of the nuclear transcription factor NF-κB, and maturation of osteoclasts [8, 13]. Bone resorption leads to bony destruction, weakening of ligaments, and consequent joint destruction secondary to contributory trauma [14]. Hyperemia through an autonomically stimulated vascular reflex causes additional periarticular osteopenia [12].

The pathogenesis of CN in the foot differs between patients with Type 1 and Type 2 diabetes. Petrova et al found a younger age at onset, a generalized reduction in bone mineral density, and more severe peripheral neuropathy in patients with Type 1 diabetes compared to those with Type 2 diabetes [15].

The progression of CN follows the stages originally described by Eichenholtz [16]. A prodromal inflammation stage with normal radiographs (Stage 0) is followed by increasing osseous destruction with radiographic evidence of osseous fragmentation and joint dislocation (Stage 1, “fragmentation”) and subsequent coalescence of fragments and absorption of fine bone debris (Stage 2, “coalescence”). Finally, chronic deformity of the foot with consolidation and remodeling of fracture fragments is observed (Stage 3, “reconstruction”).

Charcot neuropathic arthropathy severely reduces the overall quality of life and dramatically increases the morbidity and mortality of patients [17, 18]. Evidence-based guidelines for the treatment of acute CN have yet to be established. Non-operative treatment to achieve a plantigrade, stable foot and prevent recurrent ulceration is regarded as the primary treatment for acute CN feet [19, 20]. Operative treatment is often reserved for late complications, such as deep wound infection or osteomyelitis [20, 21]. Nevertheless, early reconstructive surgery in patients with an unstable foot with manifest joint subluxation or radiographic non-plantigrade foot position may provide timely restoration of the plantigrade foot [22]. In particular, unstable deformities in obese patients are sometimes difficult to brace, and these patients might benefit from primary corrective arthrodesis [23, 24]. However, despite satisfactory results, complication rates following surgery are reported to range from 10% to more than 30% [24, 25].

The aim of the present study was to compare the outcome of non-operative treatment in patients diagnosed with unilateral versus bilateral CN. Additionally, we compared outcomes of protected versus unprotected weightbearing non-operative treatment and made recommendations for non-operative therapeutic options.

## Methods

### Data acquisition

We conducted a retrospective cohort chart review study at a specialized centre for multidisciplinary treatment of foot ulcerations and deformities over an eleven-year period (2002–2012). We conducted a comprehensive search strategy of our institutional database using the keywords “Charcot” or “Neuroosteoarthropathy”. Patients older than 18 years with confirmation of CN by magnetic resonance imaging (MRI) (i.e., soft tissue edema, joint effusion, and/or subchondral bone marrow edema of involved joints, characterized by low signal intensity on T1-weighted images and high signal intensity on T2-weighted images) at the beginning of treatment were included in the study. Only patients with a diagnosis of Type 1 or Type 2 diabetes with peripheral neuropathy recorded in their charts were included. Individuals treated with immunosuppressive or osteoactive medication (i.e., bisphosphonates) and patients with osteodestructive bone pathologies, i.e. bone tumors, were excluded. Patients with diagnosed osteomyelitis or idiopathic osteoarthropathy were also excluded.

The following data were collected from the patient records: age, gender, affected limb, duration of diabetes, time period of protected weightbearing, date of first diagnosis of CN, recurrence of CN (i.e. new foot deformity and/or non-infectious swelling and redness), appearance of ulcer or infection, type of definitive treatment and surgical intervention for treatment of wounds and infections. This study was carried out in accordance with our institutional ethics committee’s terms of reference. Written informed consent allowing retrospective data analysis was received from all patients enrolled in the study.

### Patients

A total of 90 patients were identified (age 60.7 ± 10.6 years at first diagnosis of CN), with 101 affected feet. Eleven patients (12%) had bilateral CN.

The study included 22 (24%) women and 68 (76%) men, with 28 and 73 affected feet, respectively. Of the 79 unilaterally affected patients, 39 were left feet and 40 were right feet (Table 1).

**Table 1:** Baseline patient characteristics

Characteristic	Number
Patients (*n* = 90)	
Age at initial diagnosis (years), mean ± SD	60.7 ± 10.6
Follow up period (years), mean ± SD	3.7 ± 3.8
Gender, female/male, n	22/68
Bilateral patients (*n* = 11) gender: female/male, n	6/5
Comorbidity, *n* (%)^a^	
• Diabetes	90 (100)
• Peripheral vascular disease^b^	14 (16)
• Obesity (BMI > 25 kg/m2)	12 (13)
Affected feet (*n* = 101)	
Female/male, n	28/73
Left/right (in unilaterally affected patients, *n* = 79), n	39/40
Location of CN^c^ (*n* = 101 ft)	
Zone 1 (distal and proximal interphalangeal joints, metatarsophalangeal joints)	10 unilateral, 2 bilateral
Zone 2 (tarsometatarsal joints (Lisfranc)	25 unilateral, 10 bilateral
Zone 3 (naviculo-cuneiform joints, talonavicular joint; cacaneocuboid joint)	8 unilateral, 5 bilateral
Zone 4 (ankle joint, subtalar joint)	3 unilateral, 0 bilateral
Zone 5 (calcaneus)	2 unilateral, 0 bilateral
Zones 1 + 2	6 unilateral, 0 bilateral
Zones 2 + 3	1 unilateral, 0 bilateral
Zones 2 + 4	19 unilateral, 5 bilateral
Zones 3 + 4	3 unilateral, 0 bilateral
Zones 4 + 5	2 unilateral, 0 bilateral

*BMI* body mass index

^a^Some patients had multiple comorbidities

^b^Confirmed with ankle-brachial index (ABI) <0.9

^c^Based on Sanders and Frykberg Anatomic Classification [26]

CN was staged according to the Eichenholtz classification [16] by the treating clinician. In unilaterally affected patients, 6 (8%) were treated in the prodromal period (stage 0), 49 (61%) demonstrated acute stage 1 CN at the time of first diagnosis, 16 (21%) were diagnosed and treated in the coalescence stage 2, and 8 (10%) were treated for chronic stage 3 CN [16]. In bilaterally affected patients, 3 ft (14%) were treated in the prodromal period, 12 ft (54%) in the acute stage, 5 ft (23%) were diagnosed in stage 2, and 2 ft (9%) were diagnosed in stage 3. Based on Sanders and Frykberg Anatomic Classification [26], CN was located in the forefoot in 20% of feet, the midfoot in 67% of feet, and the hindfoot in 13% of feet in unilaterally affected patients (Table 1). In bilaterally affected patients, CN was located in the forefoot in 9% of feet and the midfoot in 91% of feet.

### Non-operative treatment modalities: protected weightbearing

**Fig. 1:**
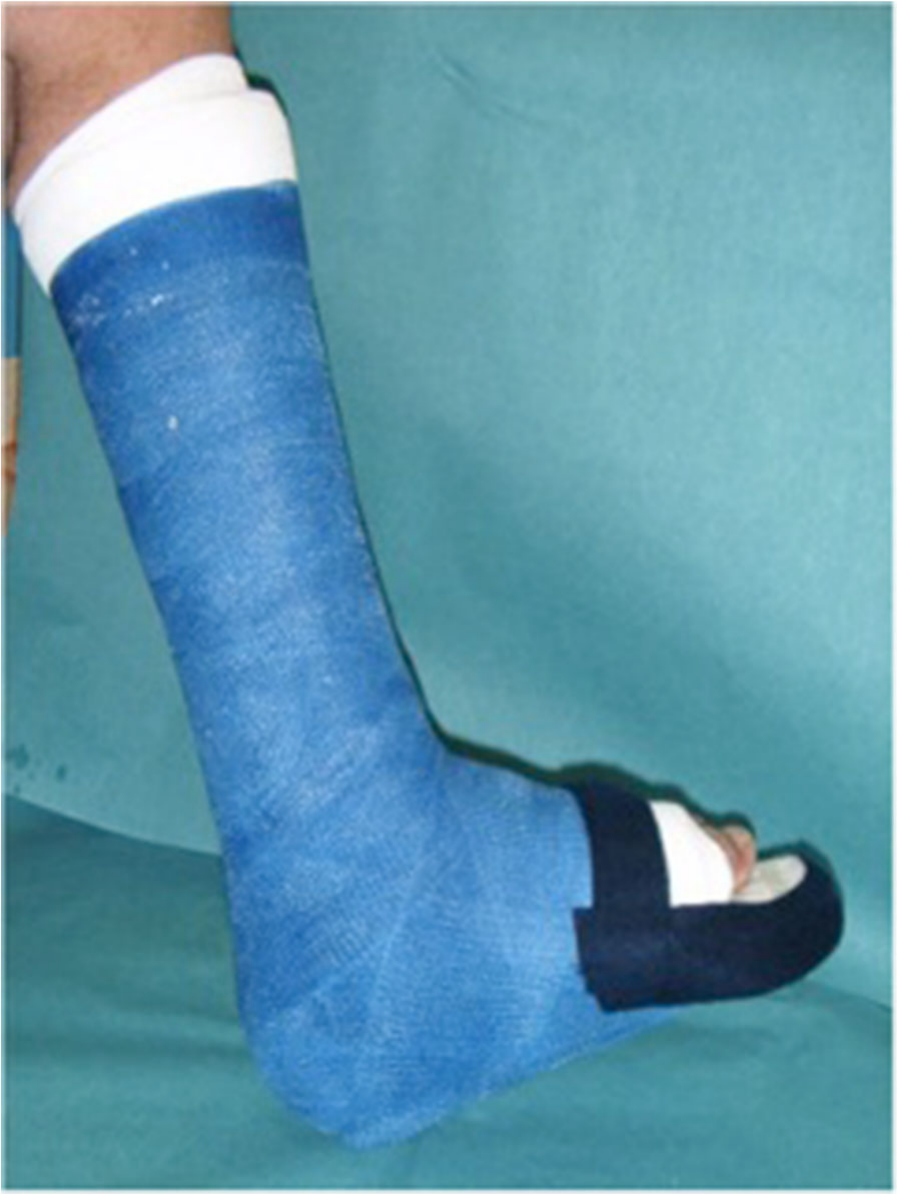
Example of a total contact cast (TCC), consisting of a properly cushioned, custom-made, rigid, fiberglass boot

**Fig. 2:**
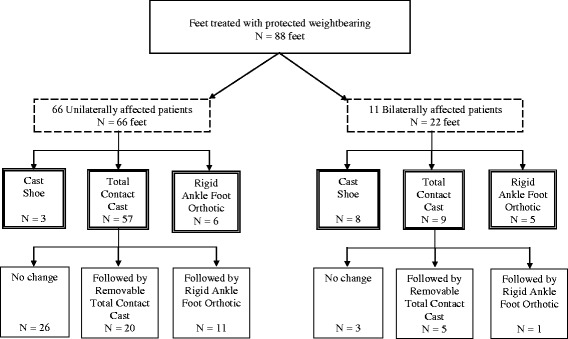
Flow chart of protected weight-bearing treatment regimen (*n* = 88 ft)

Sixty-six unilaterally affected patients were treated with protected weightbearing. Of these, 57 ft (87%) were initially supplied with a total contact cast (TCC), consisting of a properly cushioned, custom-made rigid fiberglass boot (Fig. 1). Patients were allowed to bear weight as tolerated with the aid of two crutches, if needed. Immobilization consisting of bed rest or limitation in walking activity was not required. The TCC was followed by a removable TCC (rTCC) in 20 ft (30%) or a custom-made orthosis in 11 ft (17%) when symptoms like redness and warmth had decreased sufficiently based on visual inspection and palpation and no ulcer or infection was detected (Table 2, Fig. 2). Cast changes were made at least every second week by a professionally trained technician. Patients were seen every three to four weeks in the outpatient clinic of our institution for follow-up.

**Table 2:** Protected weight-bearing treatment: initial regimen and cast types used

Cast/Shoe Type	Unilaterally affected feet (*n* = 66) N (%)	Bilaterally affected feet (*n* = 22) N (%)
Cast shoe	3 (5)	8 (35)
TCC only	26 (39)	3 (14)
TCC followed by rTCC	20 (30)	5 (23)
TCC followed by	11 (17)	1 (5)
Orthotic/AFO		
Orthotic (AFO)	6 (9)	5 (23)
Time to shoes, weeks (mean ± SD)	20 ± 44	35 ± 28

*TCC* Total Contact Cast, *rTCC* removable Total Contact Cast, *AFO* rigid Ankle Foot Orthotic

Eleven patients were affected bilaterally. Nine patients received the same initial treatment on both feet: 4 received TCCs, 3 received cast shoes, and 2 received orthotics (Table 3). The remaining two patients had different stages of CN in each foot: one patient was treated with a TCC on one foot and a cast shoe on the other; the other patient was treated with an orthotic on one foot and a cast shoe on the other. In the 9 ft that were supplied with a TCC, this was followed by a rTCC in 5 ft and a rigid ankle-foot orthosis (AFO) in 1 ft. Some patients were treated with a cast shoe containing a custom-made insole because of a lack of compliance and difficulty with conducting activities of daily living with two casts.

**Table 3:** Protected weight-bearing treatment regimen in bilaterally affected patients

Patient	Left Foot	Right Foot
1	Orthotic (AFO)	Orthotic (AFO)
2	Cast shoe	Cast shoe
3	TCC followed by removable TCC	TCC followed by removable TCC
4	TCC	Cast shoe
5	Cast shoe	Orthotic (AFO)
6	Cast shoe	Cast shoe
7	TCC	TCC followed by removable TCC
8	TCC followed by removable TCC	TCC followed by orthotic (AFO)
9	Orthotic (AFO)	Orthotic (AFO)
10	Cast shoe	Cast shoe
11	TCC	TCC followed by removable TCC

*TCC* Total contact cast

**Fig. 3:**
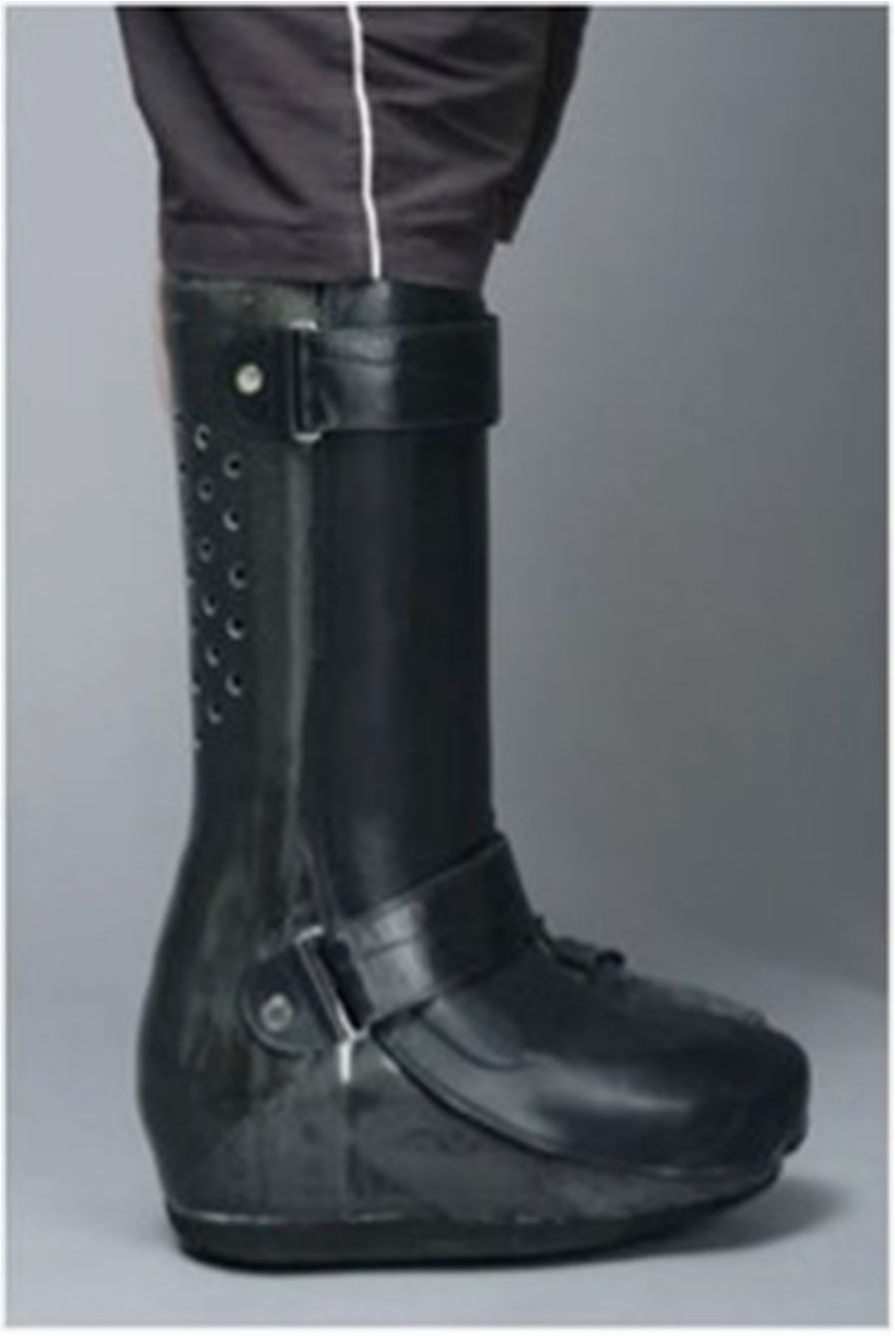
Example of a rigid ankle-foot orthosis (AFO)

A rigid AFO (Fig. 3) was used as initial treatment in 6 (9%) unilaterally affected patients and 5 ft of bilaterally affected patients. It was only used in cases of unstable foot alignment, or if the patient was unable to come to the hospital for follow-up.

In patients initially treated with protected weightbearing, definitive treatment was initiated when bony consolidation was observed. In cases of minor deformity, orthopedic shoes were fitted with custom-made insoles (33 ft (42%) in patients with unilateral CN; 7 ft (32%) in patients with bilateral CN). In cases of substantial foot deformity at the reconstruction stage, custom-made orthopedic shoes were built for 39 ft (49%) of unilaterally affected patients and 12 ft (55%) of bilaterally affected patients and an orthosis was built for 5 and 3 ft, respectively. Following definitive treatment, patients were seen every six to eight weeks, which was extended to six-month follow-up intervals with a positive clinical development.

### Unprotected weightbearing

Unprotected weightbearing was defined as orthopedic shoes with custom-made insoles and custom-made orthopedic shoes. Thirteen patients (20%) with unilateral CN refused treatment with a cast and were therefore supplied with a non-protected regimen. Of these, orthopedic shoes with custom-made insoles were provided for eight patients, and five patients received a custom-made orthopedic shoe.

Magnetic resonance imaging was used in all patients to detect remaining inflammation six months following non-operative treatment and to rule out deep soft tissue infections. The incidence of ulcer, infection (i.e., positive microbiologic culture of a deep wound biopsy), recurrence of CN and amputation was recorded in the patient charts during the follow-up visits.

The mean duration of follow-up was 45 months (range 1–208 months).

### Statistical analysis

The start and duration of protected weightbearing, as well as the type of initial treatment, were determined as potential factors influencing the appearance of ulcers and rate of infections, and therefore were considered to be prognostic factors of the disease. All data from patient records were exported into an Excel database (Microsoft Corp, Redmond, WA). For statistical analysis SPSS 17.0 software (SPSS Inc, Chicago, IL) was used. For detection of differences between groups, Chi-square or Fisher’s exact test was conducted. Significance level was defined as *P* < 0.05.

## Results

### Protected weightbearing period

Patients with unilateral CN had a shorter time period for protected weightbearing treatment (20 ± 21 weeks) compared to bilaterally affected patients (22 ± 29 weeks). In patients with unilateral CN, men had a longer period of protected weightbearing (24 ± 22 weeks) than women (16 ± 15 weeks), but the difference was not statistically significant (*p* = 0.88).

### Ulcer and infection

Outcomes following a protected weightbearing treatment regimen were compared in unilaterally and bilaterally affected patients. Five of 66 ft (8%) of unilaterally affected patients developed new ulcers during the treatment protocol. In contrast, in the bilaterally affected group, 9 of 22 ft (41%) developed new ulcers during the treatment protocol, indicating a significantly higher incidence of ulceration following a protected weightbearing in bilaterally affected patients compared to unilaterally affected patients (*p* = 0.004) (Table 4).

**Table 4:** Outcome parameters in 66 ft of patients with unilateral CN compared to 22 ft of 11 patients with bilateral CN, following a protected weightbearing treatment

	Unilaterally affected (*n* = 66)		Bilaterally affected (*n* = 22)		*P*
	Baseline	Follow-up	Baseline	Follow-up	
Ulceration, n (%)	16 (24)	21 (32)	4 (18)	13 (59)	.004^a^
- Incidence:		5 (8%)		9 (41%)	
Soft tissue infection, n (%)	2 (3)	3 (5)	1 (5)	2 (9)	
- Incidence		1 (2%)		1 (4%)	

^a^Pearson chi-square/Fisher’s exact test

In patients with unilateral CN (Table 5), new ulcerations developed in 5 of 66 ft (8%) with a protected weightbearing treatment, compared to 4 of 13 ft (31%) in patients without a protected weightbearing regimen (*p* = 0.036), indicating a significantly higher incidence of ulceration in an unprotected weightbearing regimen.

**Table 5:** Outcome parameters in unilaterally affected individuals, stratified by protected weightbearing treatment versus unprotected, full weightbearing regimen

	Protected weightbearing (*n* = 66)		Unprotected weightbearing (*n* = 13)		*P*
	Baseline	Follow-up	Baseline	Follow-up	
Ulceration, n (%)	16 (24)	21 (32)	2 (15)	6 (46)	.036^a^
- Incidence		5 (8%)		4 (31%)	

^a^Pearson chi-square/Fisher’s exact test

Thirteen patients (16%) in the unilaterally affected group received oral antibiotic treatment for a suggested soft tissue infection with signs of elevated blood infection (CRP > 5 mg/l). Blood tests (CRP levels) were only performed in a clinically suspicious situation, i.e., when a patient presented with redness and warmth of the foot, to discriminate infection from active Charcot arthropathy. Seven of 11 patients (64%) with bilateral CN were treated with prophylactic oral antibiotics for suspected soft tissue infection in one of their feet (i.e., 7/22 ft; 32%). Empiric oral antibiotic therapy was initiated and, if deep wound biopsies were taken intraoperatively, the patient was either switched to the precise oral or intravenous antibiotic therapy according to culture results and sensitivity testing, or therapy was discontinued in the case of negative culture results.

A total of 60 surgical wound procedures were performed in 50 ft during the follow-up period (Table 6). Wound debridement was performed for superficial ulcerations (*n* = 47). In the case of deep wound infection, deep surgical wound treatment with removal of sequestrum and wound lavage were performed (*n* = 13). Osseous prominences causing recurrent ulcerations were treated by exostosectomy (*n* = 4). All surgical procedures were performed by the head of the department for multidisciplinary treatment of foot ulcerations and foot deformities, or by an experienced consultant. Although bilaterally affected patients demonstrated higher rates (5 ft, 23%) of deep surgical wound therapy compared to unilaterally affected patients (8 ft, 10%), the rates were not significantly different statistically.

**Table 6:** Surgical procedures performed for wound healing in 79 ft of patients with unilateral CN compared to 22 ft of 11 patients with bilateral CN

Procedure	Unilateral CN (*n* = 79)	Bilateral CN (*n* = 22)
Wound debridement for superficial ulceration	33 (42%)	14 (64%)
Deep surgical wound treatment (i.e., removal of sequestrum and wound lavage)	8 (10%)	5 (23%)
Exostosectomy of osseous prominence	3 (4%)	1 (5%)
Amputation	3 toe	4 toe
	1 forefoot (Lisfranc)	
	2 below the knee	
	Total: 6 (8%)	Total: 4 (18%)

### Amputation

Three toe, 1 forefoot (Lisfranc), and 2 below-the-knee amputations were performed in unilaterally affected patients, and 4 toe amputations were required in bilaterally affected patients (Table 4). There were no statistically significant differences in the rates of amputation or recurrence of CN for the different treatment modalities.

## Discussion

The outcome of non-operative treatment in 90 patients (101 ft) diagnosed with CN was assessed at a mean follow-up of 48 months (range 1–208 months). The average age of patients in our investigation was 60.7 (±10.6) years, which corresponds to previously published studies [27–29].

Treatment in an orthopedic device, such as a TCC to minimize mechanical forces on the bone, achieve a plantigrade, stable foot and prevent recurrent ulceration, is considered to be an important strategy in acute CN [6, 24, 27, 30–34]. Yet, there is a wide range of recommendations concerning initial treatment. Some authors suggest non-weightbearing treatment during the first months to stop progression of deformity [27, 29, 34, 35]. In particular, they suggest that weightbearing should be prevented during the inflammation stage, to “cool down the foot” because of the risk that the unstable foot will continue to fracture [10, 35]. Frykberg et al recommended a non-weightbearing period of 8–12 weeks to avoid trauma to the affected foot [34]. Other investigators allowed weightbearing when the foot was placed in a cushioned device [10, 36–38]. De Souza et al showed that protected weightbearing in a TCC does not initiate new foot ulcers in the treatment of a Charcot foot [10]. Weightbearing does not appear to negatively affect the outcome in treatment of acute CN, as long as the foot is protected by a professionally manufactured TCC. Initial treatment in our institution consisted of a custom-made, properly cushioned, rigid plaster boot (TCC) or of a rigid ankle-foot orthosis, with weightbearing allowed as tolerated. This treatment regimen was associated with a lower incidence of ulcerations (8%) compared to an unprotected weightbearing regimen (31%) in patients with unilateral CN (*p* = 0.036). Even overweight patients with stage 1 CN according to Eichenholtz, treated with a TCC that permitted full weightbearing, successfully progressed into therapeutic footwear after an average time of 12 weeks [37]. Furthermore, non-weightbearing treatment may have an unfavorable consequence on the contralateral, unaffected limb in patients with CN, due to increased stress [30]. Clohisy et al reported that the time period to affect the contralateral limb is longer (12 months) in patients with weightbearing treatment compared to a weight-off regimen (4.5 months) [39]. Our investigation showed that the incidence of foot ulcers is higher in bilaterally affected patients compared to unilaterally affected individuals (*p* = 0.004). We therefore recommend a weightbearing treatment in a TCC in an acute stage of CN to prevent, or at least defer, progression of the disease with its complications to the contralateral side.

The alternative to a TCC is a pre-fabricated removable walker cast (i.e. Aircast®, DJO Global, Cal, USA or Vacoped®, OPED AG, Cham, Switzerland), which has the advantage of much lower costs compared to a regularly changed custom-made TCC. Use of a TCC for diabetic foot ulcers compared to a removable cast walker or half-shoe showed higher healing percentages and a shorter healing time for the TCC [2, 10, 20].

Although a 6% risk for development of pressure ulcers with the TCC has been reported, the rate of permanent sequelae from cast-related injuries is low (0.25%), and the TCC was rated as a safe modality for protected weightbearing and immobilization of the neuropathic foot [13, 40].

The major disadvantage of a removable, non-custom tailored device is a diminished compliance due to the easy removability of the device by the patient [41, 42]. This may lead to increased local pressure on the skin and, in combination with insensibility, contains an increased risk of ulceration. Compliance with wearing prescribed footwear is low [42, 43]. Only 28% of CN patients wore their removable walker brace full time (23.5 h/day), and non-compliance was shown to lead to a longer bracing period (29 ± 19 weeks) [43]. An important attribute of the TCC is that it is not easily removable and therefore has the advantage to enhance compliance [41]. It also seems to curtail activity, which reduces the number of stress cycles on vulnerable skin [41].

The protected weightbearing treatment period in a TCC in our investigation was 20 ± 21 weeks for unilaterally affected patients and 22 ± 29 weeks for bilaterally affected subjects. Our average duration of treatment corresponds to the findings of Armstrong et al with a period of 18.5 ± 10.6 weeks and Christensen et al of 20.1 ± 3 weeks [27]. However, they reported a re-casting of the unprotected extremity for a mean of 11.2 weeks in cases of exacerbation or recurrence of CN after reloading, i.e., upon initial cast removal [27, 44]. Bates et al treated 34 patients with a TCC and 12 individuals with a removable cast walker in the presence of contraindications for a TCC for 11 (range: 8 to16.7) months, and 33% had to extend their treatment period to a total duration of 20 (range, 15 to 21) (Bates M, Petrova NL, Edmonds ME: How long does it take to progress from cast to shoes in the management of Charcot osteoarthropathy? Diabetes Foot Study Group of the EASD, unpublished) months due to recurrence of inflammation. In our experience, a protected weightbearing regimen should be maintained as long as signs of inflammation such as redness and warmth are present. In the case of inconclusive clinical signs, we recommend performing an MRI to exclude residual inflammatory sites.

In our assessment, we found a shorter period until definitive treatment was initiated (mean 5 months, range 1–65 months), compared to previously published investigations. Game et al reported a duration of treatment to resolution (mobilized in orthotic or normal shoes) of 10 months (range 2–40 months) in a multicenter, web-based observational study of 288 cases in the UK. Armstrong et al suggested a time to footwear of 7 ± 3.6 months in 55 patients with CN [44, 45].

Surgical treatment was performed in cases of chronic ulceration or soft tissue infection to avoid amputation of the limb. In our experience, open surgery on an inflamed CN foot often ends in disastrous results due to infections, bone resorption, or implant loosening [46]. Therefore, it is essential to find the correct timing for such an intervention.

Nevertheless, there are situations where stability of the foot can only be achieved through operative intervention. In these situations, the circular Ilizarov fixator is an excellent treatment option because of the ability to correct multiplanar deformities [47]. Other investigators provide early reconstructive surgery in patients with advanced instability of the foot. Intervention was associated with a benefit compared to secondary operations after non-operative treatment concerning a stable, ulcer- and infection free situation [22]. The goal in CN treatment is a stable foot either by a multi-level arthrodesis or a firm fibrosis, which can be fitted with a custom-made shoe [23, 24]. El-Gafary et al treated 20 patients with CN at Eichenholtz stage 2 and presence of joint subluxations or deformities with repositioning and stabilization by application of an Ilizarov frame with restricted weightbearing [48]. They reported good clinical outcomes with a time to arthrodesis of 18 weeks (range 15–20 weeks) [48]. However, pin site infections in these situations were frequent (15 of 20 patients).

Our study has limitations. We had no measurements of skin temperature, HbA1c, or body mass index at the onset of disease or during the follow-up period. Also, the potential impact of wounds present at initiation of treatment on the primary outcome of new ulcer incidence is not well understood. Further limitations are the retrospective study design and the lack of precise matching of groups. Although our assessment included one of the largest samples available compared to other studies of CN, evaluation of treatment regimens for this disease would benefit from larger, prospective trials with homogenous patient cohorts. Nevertheless, our findings may assist in the decision making and treatment planning for a CN foot.

## Conclusion

In conclusion, protected weightbearing treatment in a TCC is a valuable option for patients with acute CN, with a significantly lower incidence of ulcerations compared to an unprotected treatment. Larger population studies are recommended to further support this observation.

## Abbreviations

ABI: Ankle-brachial index
AFO: Ankle-foot orthosis
BMI: Body mass index
CN: Charcot neuropathic arthropathy
CRP: C-reactive protein
HbA1c: Hemoglobin A1c
IL-1β: Interleukin-1β
MRI: Magnetic resonance imaging
NF-κB: Nuclear transcription factor
RANKL: Receptor activator of nuclear factor-kB ligand
rTCC: Removable total contact cast
SD: Standard deviation
TCC: Total contact cast
TNF-α: Tumor necrosis factor-α
UK: United Kingdom
